# Complete mitochondrial genome sequence of the broad-winged damselfly, *Mnais pruinosa* Selys, 1853 (Odonata: Calopterygidae)

**DOI:** 10.1080/23802359.2019.1667887

**Published:** 2019-09-19

**Authors:** Hisashi Okuyama, Takuya Kiyoshi, Jun-Ichi Takahashi, Yoshitaka Tsubaki

**Affiliations:** a Faculty of Life Sciences, Kyoto Sangyo University, Kyoto, Japan;; b Department of Zoology, National Museum of Nature and Science, Tokyo, Japan;; c Center for Ecological Research, Kyoto University, Kyoto, Japan

**Keywords:** Next generation sequence, damselfly, Mnais pruinosa, dark-brown-winged morphs, Saga Prefecture

## Abstract

In this study, we analyzed the complete mitochondrial genome of the *Mnais pruinosa* Selys, 1853 from Saga Prefecture, Japan. The mitochondrial genome of *M. pruinosa* was identified as a circular molecule of 15,494 bp, and was found to be similar to that of other damselfly species. It was predicted to contain 13 protein-coding (PCG), 22 tRNA, and two rRNA genes, as well as one A + T-rich control region. The genes *ATP8* and *ATP6* shared seven nucleotides, *ATP6* and *COIII* shared one nucleotide, *ND4* and *ND4L* shared seven nucleotides, and *ND6* and *Cytb* shared one nucleotide. The initiation codon ATG was found in eight genes, ATC in four, and ATT in one; the termination codons TAA, TAG, incomplete TA, and single T were observed in seven, one, two, and three genes, respectively. All the tRNA genes possessed a cloverleaf secondary structure, except for tRNA-His that lacks the TΨC loop. The average AT content of mitochondrial genome was 66.18%.

The broad-winged damselfly *Mnais pruinosa* Selys, 1853 (Odonata: Calopterygidae) is an endemic species, and is widely distributed across Japan. This males of this species exhibit wing color polymorphism (Sugimura et al. 1999): orange (or dark-brown) morphs, which are typically territorial males, and clear morphs, which are typically non-territorial sneakers. *M. pruinosa* often coexists with a closely related damselfly species, *M. costalis* Selys, 1869. In relation to the coexistence pattern of the two *Mnais* species, they show geographic variation in wing color polymorphism, body size, and habitat preference (e.g. Suzuki and Tamaishi 1982; Nomakuchi et al. 1984; Siva-Jothy and Tsubaki 1989; Okuyama et al. 2013; Tsubaki and Okuyama 2016). Therefore, *M. pruinosa* is an important model species for male color polymorphism that is linked to behavior and character displacements associated with species coexistence patterns. In this study, we reported a complete mitochondrial genome for *M. pruinosa* for the first time, and reconstructed the phylogenetic relationships among Odonata species.

We collected adult dark-brown-winged male *M. pruinosa* individuals from Saga Prefecture, Japan in May 2017. Adult damselflies were transferred immediately to 99.5% ethanol for mitochondrial DNA analysis. The specimen was stored the National Museum of Nature and Science, Japan (accession number: NSMT-I-Od-33338). Genomic DNA isolated from one damselfly was sequenced using the Illumina MiSeq platform (Illumina, San Diego, CA, USA). The complete mitochondrial genome of *M. costalis* (AP017642; Okuyama and Takahashi 2017) was used as a reference sequence. The resultant reads were assembled and annotated using the MITOS web server (Bernt et al. 2013) and Geneious R9 (Biomatters). These 13 protein-coding genes (PCGs) and two rRNA genes sequences were aligned with MEGA6 (Tamura et al. 2013). The phylogenetic analysis was performed with the maximum likelihood (ML) criterion using TREEFINDER Version of March 2011 (Jobb 2011).

The *M. pruinosa* mitochondrial genome forms a 15,494 bp-long closed loop (DDBJ accession number AP019628). It represents a typical Odonata mitochondrial genome in that it comprises 13 PCGs, 22 putative tRNA genes, and two rRNA genes, as well as an A + T-rich control region. The average AT content was 66.18%. Similar to other damselfly mitochondrial genomes, the heavy strand was predicted to have nine protein-coding and 14 tRNA genes and the light strand was predicted to contain four protein-coding, eight tRNA, and two rRNA genes. Among the 13 PCGs, the initiation codon ATG was found in eight genes, ATC in four, and ATT in one, while the termination codons TAA and TAG were observed in seven PCGs and the *ND3* gene, respectively. The incomplete stop codons TA (*COIII* and *ND4*) and T (*COII*, *ND5*, and *Cytb*) were identified. All the tRNA genes possessed a cloverleaf secondary structure; however, *tRNA-His* lacked the TΨC loop. A phylogenetic analysis of 30 Odonata taxa using 13 PCGs (Figure 1) showed that *Mnais* forms a single, distinct clade. It has been suggested that it may not be possible to distinguish between the two Japanese *Mnais* species using the only mitochondrial genome (Hayashi et al. 2005); it is therefore necessary to analyze more *Mnais* individuals.

**Figure 1.**
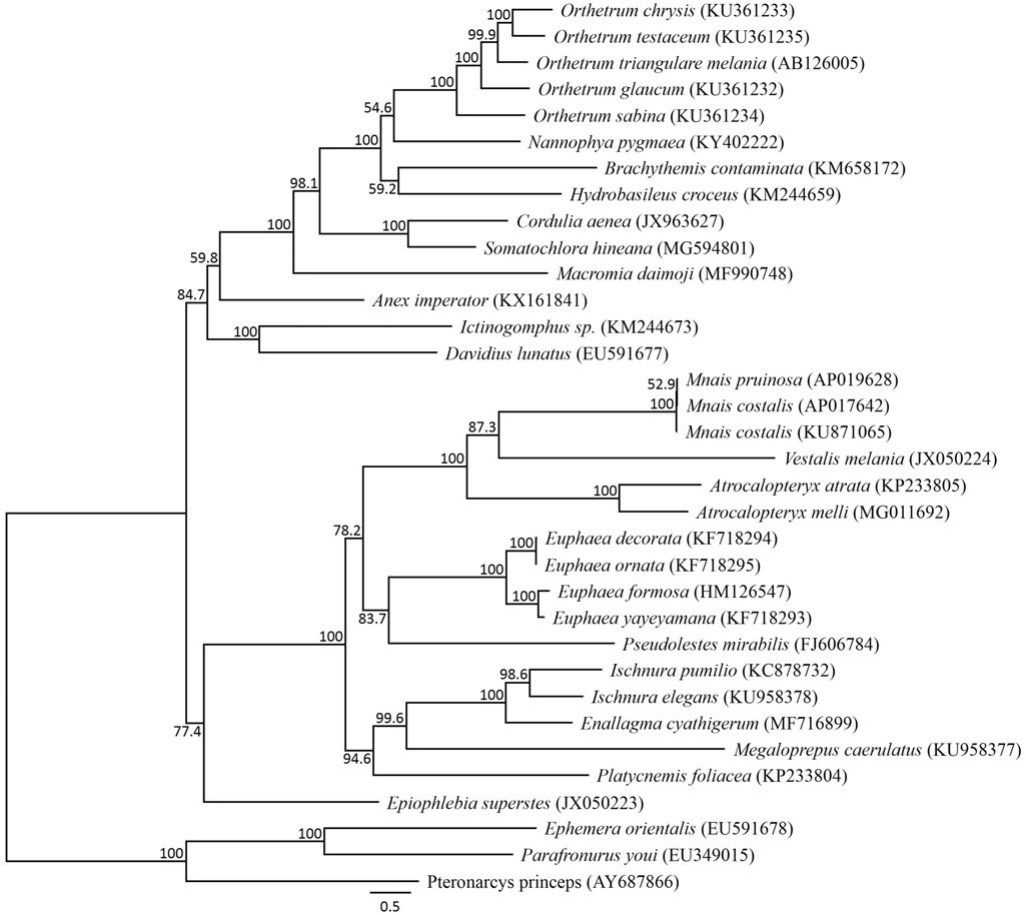
Phylogenetic relationships (maximum likelihood) of the Odonata based on the nucleotide sequences of the 13 protein-coding genes of the mitochondrial genome. Sequences from *Ephemera orientalis* (EU591678, Lee et al. 2009), *Parafronurus youi* (EU349015, Zhang et al. 2008), and *Pteronarcys princeps* (AY687866, Stewart and Beckenbach 2006) were used as an outgroup. These sequences were separated by codon positions, and for each partition, the optimal models of sequence evolution were used in the maximum likelihood method using TREEFINDER, based on the corrected Akaike information criterion. The numbers at the nodes indicate the bootstrap support inferred from 1,000 bootstrap replicates. Alphanumeric terms indicate the DNA Database of Japan accession numbers.
